# Epstein-Barr Virus as a Trigger of Autoimmune Liver Diseases

**DOI:** 10.1155/2012/987471

**Published:** 2012-05-28

**Authors:** Eirini I. Rigopoulou, Daniel S. Smyk, Claire E. Matthews, Charalambos Billinis, Andrew K. Burroughs, Marco Lenzi, Dimitrios P. Bogdanos

**Affiliations:** ^1^Department of Medicine, University of Thessaly Medical School, Viopolis, 41110 Larissa, Greece; ^2^Institute of Liver Studies and Liver Unit, King's College London School of Medicine, King's College Hospital, Denmark Hill Campus, London SE5 9RS, UK; ^3^Department of Microbiology and Parasitology, Faculty of Veterinary Medicine, University of Thessaly, 43100 Karditsa, Greece; ^4^The Sheila Sherlock Liver Centre and University Department of Surgery, Royal Free Hospital, London NW32QG, UK; ^5^Department of Clinical Medicine, Alma Mater Studiorum, University of Bologna, Sant'Orsola-Malpighi Policlinic, Padiglione 11, 40138 Bologna, Italy; ^6^Cell Immunotherapy and Molecular Immunodiagnostics, Biomedical Research & Technology, CERETETH, 41222 Larissa, Greece

## Abstract

The pathogenesis of autoimmune diseases includes a combination of genetic factors and environmental exposures including infectious agents. Infectious triggers are commonly indicated as being involved in the induction of autoimmune disease, with Epstein-Barr virus (EBV) being implicated in several autoimmune disorders. EBV is appealing in the pathogenesis of autoimmune disease, due to its high prevalence worldwide, its persistency throughout life in the host's B lymphocytes, and its ability to alter the host's immune response and to inhibit apoptosis. However, the evidence in support of EBV in the pathogenesis varies among diseases. Autoimmune liver diseases (AiLDs), including autoimmune hepatitis (AIH), primary biliary cirrhosis (PBC), and primary sclerosing cholangitis (PSC), have a potential causative link with EBV. The data surrounding EBV and AiLD are scarce. The lack of evidence surrounding EBV in AiLD may also be reflective of the rarity of these conditions. EBV infection has also been linked to other autoimmune conditions, which are often found to be concomitant with AiLD. This paper will critically examine the literature surrounding the link between EBV infection and AiLD development. The current evidence is far from being conclusive of the theory of a link between EBV and AiLD.

## 1. Introduction

Several viruses have been considered to be triggers of autoimmunity and overt autoimmune disease [1–6]. Amongst those, Epstein-Barr virus (EBV), which is the cause of infectious mononucleosis, is unique in a sense as it has been implicated in the induction of multiple autoimmune diseases [7, 8]. These include systemic lupus erythematosus (SLE), multiple sclerosis (MS), autoimmune thyroiditis (AT), rheumatoid arthritis (RA), inflammatory bowel diseases (IBD), insulin-dependent diabetes mellitus (IDDM), Sjögren's syndrome (SjS), systemic sclerosis (SSc), myasthenia gravis, and autoimmune liver diseases (AiLD) [7–20]. In fact, there are very few autoimmune diseases in which EBV has not been considered as a potential trigger of immune-mediated destruction. While in some of these diseases there is growing evidence in support of the link between EBV and autoimmunity, the link is not as strong as in others, and the pathogenic involvement of EBV is a matter of heated debate.

This paper will discuss the clinical and experimental data investigating the role of EBV in the pathogenesis of AiLD. As these diseases frequently co-occur with extrahepatic autoimmune diseases, we also discuss EBV's involvement in the pathogenesis of autoimmune manifestations seen in patients with AiLDs, as this topic has not been discussed previously in great detail. AiLDs include a heterogeneous group of disorders that affect the hepatocytes as in the case of autoimmune hepatitis (AIH) and the cholangiocytes as in the case of primary biliary cirrhosis (PBC) and primary sclerosing cholangitis (PSC). The evidence for EBV as a causative agent varies from one autoimmune liver disease to the other (Table 1). However, there does not appear to be any conclusive evidence linking EBV with the induction of AiLD.

## 2. General Aspects of Autoimmunity

It is generally supported by most researchers that the vast majority of autoimmune diseases develop from the close interaction of genes, epigenetic factors, and “multiple hits” from environmental agents [21, 22]. More than 100 types of autoimmune disease have been described so far. Individual autoimmune diseases appear rare, but it is estimated that ~5–20% of the North American population has at least one autoimmune disease [23]. 

The aetiology of autoimmune disease is complex, involving immunological, genetic, and environmental factors. The extent to which these factors are implicated differs from one disease to the next. Monozygotic concordance rates below 50% support the notion that environment as well as genetics are closely involved [24–28]. As well, epidemiological studies on populations with the same or similar genetic or ethnic background living under different conditions or migrating to different places have reported different incidence rates of autoimmune disease. The development of autoimmune disease as a result of exposure to these factors can be mediated by a variety of mechanisms such as T-cell dysregulation, nonspecific activation of the immune system, release of cryptic antigens, altered structure or expression of autoantigens, antiapoptotic effects on autoreactive cells, molecular mimicry and immunological cross-reactivity, to name a few [1, 29–33]. In recent years, large genetic studies known as genomewide association studies (GWASs) have discovered several gene associations with various autoimmune disorders [34]. Although GWASs have been pivotal in revealing specific genetic influences, exposures to infections are also likely to participate in the breakdown of immunological tolerance and the development of full-blown autoimmune disease [34, 35]. In fact, some of the genes that confer susceptibility to autoimmunity may also influence the penetrance of infectious agents and the appearance of recurrent infections with viruses or microbes implicated in the induction of autoimmunity. Epidemiological studies have helped us to identify infectious and noninfectious environmental agents (such as xenobiotics, chemical compounds, radiation, vaccines) [35]. The prevailing notion from the GWAS is that multiple genes are needed to induce autoimmunity [34]. Epidemiological, clinical, and experimental studies have also revealed that several infectious triggers are most likely needed to provoke immune-mediated processes that lead to autoimmunity. The additive effects of these agents and their interplay with susceptible genes remain poorly understood. In fact, on several occasions the same infectious agents that induce autoimmunity may also be responsible for the abrogation of autoimmune phenomena in another clinical setting.

Against this background which suggests that the induction of autoimmune disease is a complex, multifactorial process involving a plethora of genetic, infectious, and environmental agents working in concert, there are studies to suggest that a single virus may possess a unique property to induce generalised autoimmunity. These studies single out EBV as the most likely candidate with such properties [7, 8, 36].

The ideal virus which can induce autoimmunity would need to explain disease-specific features frequently seen in the majority of autoimmune disorders. For example, involvement of this virus would need to provide clues as to why some autoimmune diseases run a subclinical course of several years, with diagnosis of the disease being made after the target organ has been damaged. Such cases are found in a significant proportion of patients with AiLD, AT, and IDDM, for example. Viral-induced autoimmunity will also need to explain the relapsing-remitting course of MS, RA, ulcerative colitis, PSC and AIH [1, 4–6]. As most autoimmune diseases are characterised by non-organ and organ-specific autoantibodies, the virus in question will need to be able to provoke the induction of a wide variety of autoantibody specificities [2]. It would also need to explain the female preponderance of most autoimmune diseases and the tendency of these diseases to be unmasked during the postmenopausal or postpartum period [21].

## 3. EBV as a Trigger of Autoimmunity

EBV (also known as human herpes virus 4) infection is widespread, with 98% of the world's population being infected [8]. Its genome is composed of a 172 kb double strand of DNA [8]. Primary infection with EBV is followed by viral replication in nasopharyngeal epithelia and B cells, followed by a latent stage in B cells [8]. There are several features of EBV which make it an appealing culprit in the pathogenesis of autoimmune disease [7, 8, 36]. These include its high prevalence worldwide, its persistency throughout life in the host, its ability to alter the immune system of the host and to inhibit apoptosis, as well as the fact that infection precedes the onset of disease symptoms [8]. 

Amongst those, the most intriguing characteristic of EBV is its ability to infect, activate, and latently persist in B lymphocytes. *In vitro* systems of resting B cells infected with EBV showed that these cells proliferate without the need for “T-cell-dependent help” [37]. Also, *in vitro* studies have shown that EBV-transformed B lymphocytes can induce production of monoclonal antibodies targeting various organ-specific autoantigens [38]. Most of the EBV-infected proliferating B cells act as targets of virus-specific CD8+ T cells and are eliminated from the circulation, however memory B cells can persist for life in infected individuals [37]. 

Most studies implicating EBV as a trigger of autoimmune diseases are based on findings indicating high titers of EBV-specific antibodies in patients compared to controls [3]. Additionally, a higher proportion of EBV-infected cells with elevated EBV loads are found in peripheral blood lymphocytes compared to other pathological or normal controls. Diseases with an increased risk of lymphoma like primary SjS, RA, and SLE are considered to be more prone to be induced by EBV compared to others with a risk of lymphoma comparable to that of the control population.

## 4. EBV and AIH

AIH is an inflammatory liver disease primarily affecting women and is characterised by elevated transaminase levels, the presence of specific autoantibodies, raised IgG, and interface hepatitis on histology [39–44]. Although the aetiopathogenesis of AIH is unclear, several autoimmune pathways have been proposed which are largely based on autoreactive CD4+ T lymphocytes recognizing a liver-specific autoantigen [45–47].

AIH can be divided into AIH type 1 or type 2, based largely on distinct autoantibody profiles [48, 49]. AIH type 1 affects all ages while AIH type 2 is practically limited to the paediatric population. Antinuclear antibody (ANA) and smooth muscle antibody (SMA) characterize type 1, while antibodies to liver kidney microsomal antigen type 1 (anti-LKM1) and anti-liver cytosol type 1 antibodies (anti-LC1) are characteristics of type 2 [39, 41, 44, 48, 49]. Other autoantibodies include antisoluble liver antigen/liver pancreas antibody (SLA/LP) and antibodies against the asialoglycoprotein receptor (ASGP-R) [39, 41, 44, 48, 49].

Conventional treatments of AIH include immunosuppression with prednisolone, with or without azathioprine [39, 40, 50]. Most patients show a good response to treatment, which should be initiated soon after diagnosis. Left untreated, AIH progresses to cirrhosis and liver failure requiring liver transplantation [39, 40, 50]. The prevalence of AIH is estimated to be 1.9 to 20 per 100,000, but this is likely underestimated [39, 40, 50]. The diseases is characterised by a female predominance with 75% of patients being female and a family history of autoimmune disorders which is found in 40% of cases [39–44]. 

AIH may present acutely in approximately 40% of cases and may resemble acute viral hepatitis [39–44, 51]. The remaining cases may have an insidious onset presenting with less acute illness, characterized by relapsing jaundice, fatigue and weight loss over months to years [39–44, 51]. Other causes of chronic liver disease such as viral hepatitides, Wilson's disease, drug-induced hepatitis, alcoholic hepatitis, as well as other infrequent causes of chronic liver disease must be excluded [39–44, 51]. The aetiopathogenesis of AIH is poorly understood, but there is some evidence to suggest that a numerical and functional impairment of T-regulatory cells may be involved [45, 52–55]. In addition, a variety of genetic and environmental factors are involved in the development of the disease, including viruses. AIH following an acute viral infection has been strongly associated with hepatitis A virus (HAV) and hepatitis B virus (HBV), but several cases report the development of AIH following EBV infection [20, 56–58]. A prospective study conducted by Vento et al. followed the relatives of 13 AIH patients, in order to determine whether these relatives developed serological or clinical evidence of AIH [20]. Infectious mononucleosis developed in 7 of the 13 patients following EBV infection, with AIH developing within 4 months of this infection in 2 of the 7 individuals [20]. 

A case report by Kojima et al. [57] also describes the association of AIH with EBV infection. That case involved a 73-year-old man presenting with fever, lymphadenopathy, granulocytopenia, thrombocytopenia, ascites, pleural effusion, liver injury, and a skin rash [57]. Antibodies to EBV were detected, and circulating lymphocytes were positive for the EBV genome [57]. ANAs were also present, and the histological features of a liver biopsy resembled those seen in AIH [57]. A paediatric case of a 5-year-old with AIH-like histopathological features induced after EBV infection has also been reported [58]. These case reports are suggestive of EBV inducing paediatric as well as adult forms of AIH type 1, but it still remains unclear whether there is a true link or it is due to the rarity of these cases and the lack of direct evidence associating the two conditions. Due to the universal exposure to EBV infection, an increased number of studies reporting induction of EBV-induced AIH would be expected, but this appears not to be the case.

One group reports the case of fatal chronic active EBV infection mimicking AIH [59]. That 22-year-old female presented with pyrexia, pancytopenia, and liver dysfunction. Her previous medical history included a diagnosis of AIH six years previously, after presenting with liver dysfunction, low-grade fever, and mild hepatosplenomegaly [59]. Liver biopsy on admission indicated chronic active EBV infection, including *in situ* hybridization which demonstrated lymphocytes and Kupffer cells positive for EBV-encoded small RNA-1 [59]. Serological markers for EBV were also raised. She was commenced on prednisolone and cyclosporine [59]. Ten months after diagnosis, the patient died due to liver failure and virus-associated hemophagocytosis [59]. Retrospective examination of serum samples taken at the previous diagnosis of AIH showed high titres of EBV antibodies and EBV-DNA was detected by PCR [59]. Those authors highlight that hepatic involvement of chronic active EBV infection should be included in the list of differential diagnoses, in cases of liver dysfunction with clinical and biochemical features of AIH [59]. In contrast is a case report by Nakajima et al. [60] which describes the case of a ten-year-old female, admitted due to cholestasis and elevated liver enzymes for 2 months. EBV-DNA was found to be raised in the peripheral blood mononuclear cells [60]. She had hypergammaglobulinemia and was positive for ANA and SMA [60]. Liver histology demonstrated interface hepatitis, dense mononuclear cell infiltrates, and mild fibrosis, but was negative for EBV by *in situ* hybridization [60]. Those authors highlight that liver histology was essential to differentiate AIH from EBV hepatitis [60]. Another case of EBV as a trigger of AIH has been reported by Cabibi et al. [61]. A 31-year old male was admitted with jaundice, generalised fatigue, and abnormal liver biochemistry tests [61]. Anti-HAV, anti-HBV, and anti-HCV were negative, as were ANA, SMA, and AMA [61]. Antibodies to hepatitis E were positive, a diagnosis of acute hepatitis type E was made, and the patient's condition progressively improved [61]. Over one year later, the patient had markedly raised aminotransferase and a relative lymphocytosis [61]. Anti-VCA/EBV IgM was found to be positive, but all other viral markers and autoantibodies were negative [61]. ANA became positive several months later, with ANA-HEp2 being detected at a titre of 1 : 2560 with a homogeneous pattern, which is specific for AIH. IgG anti-VCA/EBV and EBNA/EBV IgG were positive; however, EBV-DNA was negative in peripheral mononuclear cells [61]. Liver histology demonstrated chronic hepatitis, which was consistent with AIH [61]. The patient was commenced on methylprednisolone and eventually azathioprine, which were both stopped when liver biochemistry returned to normal [61].

Apart from the previous case reports, no comprehensive studies link EBV infection with the development of AIH. As well, the potential mechanisms that may be involved in pathogenic process of AIH following EBV infection, such as molecular mimicry, have not been explored extensively. At best, it can be stated that EBV induced AIH in individual cases, but overall, there is no evidence linking EBV with AIH.

## 5. EBV and PBC

PBC is a chronic cholestatic liver disease characterized by immune-mediated destruction of the small intrahepatic bile ducts [62–64]. The prevalence of PBC varies from 28 to 402 per million and appears to be rising [65–68]. The disease mostly affects middle-aged women [65]. High-titer serum antimitochondrial autoantibodies (AMAs) are pathognomonic for PBC [48, 62–64, 69–78], and its presence in asymptomatic patients is believed to be predictive of eventual disease development [79]. Approximately 30% of patients have PBC-specific ANA [48, 49, 70, 72, 74, 77, 80–82]. ANA may be present in AMA negative PBC patients, in addition to asymptomatic individuals and family members of PBC patients [83–89]. Some studies suggested that patients with PBC-specific ANA have a poorer prognosis [83, 86–98].

Presenting symptoms are often nonspecific such as pruritus and/or fatigue or can be more severe with jaundice and portal hypertension [62–64]. Most asymptomatic patients are diagnosed incidentally [99, 100]. The diagnosis of PBC is based on elevated alkaline phosphatase (ALP), the presence of serum AMA (titre ≥1 : 40), and characteristic liver histology [62–64, 101]. Medical treatment for PBC is with ursodeoxycholic acid (UDCA), which has greatly improved the life expectancy and quality of life of PBC patients [102, 103].

The pathogenesis of PBC is unclear but experimental and clinical data suggest that the disease is autoimmune in nature [104, 105]. This includes the presence of autoreactive T and B cells against specific antigens in the peripheral blood and the inflamed liver tissue of PBC patients and high-titre serum autoantibodies characteristic of the disease [69, 75, 105–114]. There is also evidence in support of infectious agents and xenobiotics mimicking the major autoantigen in PBC, PDC-E2, can induce loss of immunological tolerance and biliary epithelial-specific pathology, leading to the destruction of the bile ducts [22, 115–117].

Some studies have found an association between PBC and EBV infection [118]. Morshed et al. [119] obtained peripheral blood mononuclear cells (PBMC), liver tissue, and saliva (see the following) from PBC patients and subjected them to PCR for the detection of viral sequences. Increased EBV-DNA was found in 61% of PBMC, compared to 19% in autoimmune hepatitis, 14% in patients with liver cirrhosis of various causes, and 11% in healthy controls [119]. Liver and salivary samples also showed increased levels of EBV-DNA in PBC patients compared to pathological and healthy controls [119]. A study by Barzilai et al. [3] examined 1595 serum samples from 23 autoimmune diseases and screened these for EBV and CMV infection. Sixty nine samples were from patients with PBC [3]. Increased EBV early antigen IgG titres were noted to be raised in PBC patients compared to normal controls [3]. However, this was not specific for PBC, as other diseases such as SLE, RA, antiphospholipid syndrome, MS, polymyositis, SSc, SjS, giant cell arthritis, Churg-Strauss vasculitis, and cryoglobulinaemia were also characterized by increased EBV early antigen IgG titres [3]. 

Much like AIH, there is no evidence conclusively linking PBC and EBV. The higher prevalence of EBV is not specific to PBC but appears to be a characteristic of autoimmune disease in general. This must be taken into account when examining the prevalence of EBV in autoimmune diseases that are and those that are not concomitant with PBC. Relatively limited data are available which may suggest a role for molecular mimicry as a potential mechanism of EBV-induced PBC (see the following).

## 6. EBV and PSC

Data on the association of EBV with PSC are scarce. PSC is a chronic progressive cholestatic disease, characterized by immune-mediated destruction of the biliary epithelial cells leading to fibrosis and biliary cirrhosis. The reported prevalence of PSC varies amongst studies but has been estimated at 10 per 100,000 in Northern European populations. In their great majority, patients with PSC are young males. This condition is associated with an increased risk for cholangiocarcinoma and concurrence of inflammatory bowel disease. The diagnosis of the disease is largely based on endoscopic cholangiography and/or magnetic resonance imaging and to a lesser extent on biochemical indices of cholestasis and disease-related immunological profiling which mainly includes perinuclear antineutrophilic antibodies (p-ANCA). As for PBC, pharmaceutical management of PSC includes administration of ursodeoxycholic acid. This regimen, however, does not improve survival and curative treatment consists of liver transplantation. In the absence of cholangiocarcinoma, most patients with PSC who are transplanted have excellent rates of survival. 

Though there is no direct evidence of involvement of EBV in PSC, there are studies to implicate EBV with UC with or without concurrent PSC. Lidar et al. [120] investigated antibody serology against various infectious agents and compared their titres in patients with IBD compared to healthy controls. Antibody titres against EBV did not differ between UC and CD or between IBD and healthy controls [120]. However, an increase number of EBV-infected B lymphocytes in intestinal mucosal samples from patients with ulcerative colitis compared to healthy controls have been reported [121, 122]. It is not clear, however, how many of the UC patients with increased number of EBV-infected B lymphocytes had coexistent PSC [122]. To date, there does not appear to be strong or direct evidence linking EBV and PSC. It is not clear as to whether an association between UC and EBV infection plays a role in the association of UC with PSC.

## 7. EBV and Extrahepatic Autoimmune Manifestations in AiLD

It appears that in contrast to other autoimmune diseases, the evidence linking EBV with liver autoimmunity is not strong. There are several reasons why this may be. For example, if EBV was a predominant trigger of AiLD, we would expect to see EBV-related extrahepatic autoimmune diseases in a large proportion of AiLD patients. This does not appear to be the case for MS and SLE which have been linked with EBV but are infrequently found in AIH and PBC. However, other extrahepatic autoimmune diseases such as autoimmune thyroid disease (AiTD), SjS, and RA linked to EBV do appear as concomitant autoimmune diseases in a significant proportion of patients with AiLD.

AiTD and SjS are two of the most common concomitant autoimmune diseases to affect patients with AiLD. It has been demonstrated that increased levels of anti-EBV antibodies are present in the sera of patients with AiTD and SjS [19, 123, 124]. EBV appears to have a role in the pathogenesis of thyrotoxicosis, which may occur immediately after infectious mononucleosis, and autoimmune hypothyroidism can develop from acute EBV infection [125]. As well, EBV-infected B cells may play a role in the development of B-cell lymphoma in the thyroids of patients with AiTD [126]. As mentioned, saliva samples from patients with PBC have been shown to have high titres of EBV [119]. This is of interest given that increased EBV shedding from the oropharynx has been observed in SjS patients [124], and SjS or *Sicca* symptomatology is frequently observed in patients with PBC.

RA is also commonly found in patients with AiLD, and EBV infection also appears to be a feature of RA. Increased anti-EBV antibodies have been found in the sera of RA patients [127], and another study has found a ten-fold increase of EBV-DNA in the peripheral blood mononuclear cells of RA patients compared to normal controls [128]. There remains, however, some controversy as to the role of EBV in the pathogenesis of RA. Using highly sensitive *in situ* hybridization techniques to detect EBV-encoded small nuclear RNAs (EBERs) in biopsy samples of synovial membranes of RA patients, Niedobitek et al. [129] concluded that there was insufficient evidence to support the role of EBV in the pathogenesis of RA. However, 19% of their cohort of 37 RA patients did have EBERs, which were not found in any of the controls from patients with non-RA joint disease [129]. EBERs were expressed by B cells and plasma cells [129]. Finally, EBV-specific CD8+ T cells are abundant in the inflamed joints of RA patients, although this is also found in CMV-infected joints [130].

## 8. The Role of Molecular Mimicry between EBV and Self in AIH

The mechanisms by which microbial agents may induce autoimmune disease are currently unknown, although molecular mimicry between microbial and self-peptides has been indicated [56, 83–85, 106, 131–135]. Several studies have provided evidence for T cell or antibody cross-reactivity between EBV and self-antigens [136–140]. This includes myelin basic protein in MS [136, 139], La antigen in SjS [137], SmD in SLE [138], and MHC-derived peptides in oligoarticular juvenile idiopathic arthritis [140].

As anti-LKM-1 antibodies of patients with type 2 AIH recognize a short epitopic region on CYP2D6 spanning amino acids 254–271, attempts have been made to identify viral triggers that could initiate virus/self-cross-reactive immune responses. Following original reports demonstrating amino acid similarities between CYP2D6_254–271_, HCV, and HSV-1 [141, 142], researchers from King's College London identified additional mimics originating from human adenovirus, human cytomegalovirus, and EBV [135, 143].

The CYP2D6_259–271_ mimicking sequences from EBV (Table 2) originated from the nuclear protein EBNA and the virion protein BVRF1. The EBNA mimic shared with CYP2D6, the hexameric—PAQPP—motif which is the core region, is also shared between CYP2D6 and HSV-1. Despite its high degree of homology with the self-epitope, the BVRF1 EBV peptide was totally unreactive in anti-CYP2D6 254–271 positive patients with AIH-2. The EBNA EBV mimic has been found to be a specific target of antibody responses in serum samples from patients with AIH-2 [135, 143]. 

The same group of investigators have also described a case of a girl who developed anti-LKM1 following infection at the time of liver transplantation (LT) for end-stage alpha-1 antitrypsin deficiency-related cirrhosis [144]. The girl developed overt *de novo* AIH eight years after LT. This girl had been exposed to EBV and HSV-1 prior to LT and acquired HCV and CMV infections subsequent to LT [144]. Experiments investigating reactivity to virus/self-mimicking sequences were performed in serum samples, collected between 1989 and 2000. An *in house* ELISA was used to assess cross-reactivity between CYP2D6 (252–271), the key epitope of LKM1 reactivity, and its HCV, HSV-1, CMV, and EBV viral homologues. Before LT, only an IgG response to an HSV-1 mimic was present, while the newly acquired CYP2D6_252–271_ response was accompanied by reactivity to E1/HCV, CMV, and HSV mimics after LT [144]. Reactivity to EBV mimic became detectable 2 months after LT. Reactivity to CYP2D6_252–271_ was still present when *de novo* AIH was diagnosed 8 years later. Identical LKM1 reactivity was detected eleven years after LT [144]. These data supported the notion that a CYP2D6_252–271_ crossreactive lymphocyte population, primed by HSV-1, underwent expansion following exposure to mimicking sequences of HCV, CMV, and EBV followed by production of LKM1 and ultimately AIH development [144].

## 9. The Role of Molecular Mimicry between EBV and Self in PBC

Several studies have demonstrated cross-reactivity between microbial peptides and the major epitope in PBC, known as PDC-E2 [131]. One study examined various microbial sequences for similarity with PDC-E2 [131]. Sequence similarity between the major human PDC-E2 autoepitopic region was found only with *Escherichia coli*, *Helicobacter pylori*, *Pseudomonas aeruginosa*, human cytomegalovirus and *Haemophilus influenza* [131]. No similarities were noted with EBV [131]. 

As mentioned, EBV antigens that could serve as targets of cross-reactive antibodies specific for human PDC-E2 have not been found [145]. However, other groups have examined cross-reactivity between EBV sequences and sp100, the major nuclear body autoantigen of PBC-specific anti-MND antibody responses [146]. These antibodies are found in approximately 30% of PBC patients, and those of the IgG3 subclass have been associated with a more severe disease progression [89]. Xie and Snyder [146] mapped epitopes which react with autoantibodies to sp100 (nuclear dot). Immunoblotting demonstrated that 10 of 12 sera recognized two major autoepitopes of approximately eight amino acids long [146]. These two linear epitopes share homology with several viral proteins which were found in a database, including two fusion proteins containing EBV protein sequences [146]. These sequences included the UL37 which shared homology with ND EI (epitope E1-V1), as well as EBN4 and KITH which shared homologous regions with ND EII (epitopes EII-V1 and V2, resp.) [146]. Intriguingly, the UL37 and KITH sequences showed reactivity with sera from patients with autoimmune disease [146]. These data support the hypothesis that EBV may be involved in the pathogenesis of autoimmune diseases with sp100, such as some cases of PBC.

## 10. Autoimmune Liver Diseases, EBV, and Immune Dysregulation

As noted in the familial studies conducted by Vento et al. [20, 147], the two relatives who became infected with EBV and HAV, and subsequently developed AIH, both had preexisting ASGPR suppressor-inducer T-cell defects. This has led to the hypothesis incorporating a genetic predisposition with viral infection in the development of AIH [148]. In that theory, an individual would have to have a genetic predisposition, such as a particular HLA haplotype associated with AIH and impairment of antigen-specific autoreactive regulatory cells [148]. Infection with HAV or HBV could lead to persistent T- and/or B-cell reactivity to liver-related autoantigens such as ASGPR in these individuals, leading to cytotoxicity to normal hepatocytes, and the development of AIH [148]. This theory may also be applicable to other viral infections, with T- and/or B-cell reactivity to antigens separate from the ASGPR. Several years later, it was demonstrated that a numerical and functional dysregulation in CD4+ CD25+ T-regulatory cells (Treg) is a feature in AIH patients [52–55]. Newly described Th17 cells may also be involved, but their role is currently not well defined [45]. These studies clearly indicate that the pathogenic processes involved in AIH would have to occur in an environment in which regulatory T cells do not oppose, or in which there is a dysregulation of T-regulatory cell function [52–55, 149]. However, the relevance of these results with the status of immunity specific for EBV has not been studied in detail.

IPEX syndrome is a congenital disorder characterized by immune dysregulation, caused by mutations in the FOXP3 gene of the X chromosome, which is required for the suppressive function of naturally arising CD4+ CD25+ regulatory T cells [150–152]. Defects in these pathways are therefore capable of inducing autoimmunity. Patients with IPEX syndrome are known to produce a variety of autoantibodies including AMA [153]. Tsuda et al. report the case of an 11-year-old male with IPEX, who was positive for AMA, but with no clinical or biochemical evidence of liver disease [153]. It is not known whether the AMA in that case was directed against PDC-E2, and it is unknown whether the child in that case eventually went on to develop PBC [153]. Lucas et al. report the case of a child with IPEX that developed EBV-induced lymphoma [154]. Those authors note that immunosuppressive therapy can be used to control autoimmune manifestations in patients with IPEX [154]. The 3-month-old male IPEX patient in their report was treated with rapamycin, which improved his eczema, diarrhhoea, and aphthous stomatitis [154]. Eight months after treatment, he presented with fever and tender cervical lymphadenopathy, which was later diagnosed as B-cell lymphoma with positive EBV testing [154]. These cases highlight the possibility that EBV may induce autoimmunity in genetically susceptible individuals, such as those with IPEX syndrome. It is also possible that the immunological dysregulation seen in these conditions may increase their susceptibility to EBV infection. 

A recent paper by Pender [155] presents a compelling hypothesis involving immunological dysregulation, EBV infection, and the development of autoimmunity. CD8+ T-cell deficiency is a characteristic of several autoimmune diseases and also occurs in some relatives of patients with autoimmune disease, suggesting an underlying genetic susceptibility [155]. Pender suggests that the impairment of CD8+ T cells results in the inability to control EBV infection and thus there is accumulation of EBV-infected, autoreactive B-cells in a variety of target organs, with clonal expansion of these cells, and the development of ectopic lymphoid follicles [155]. Within the target organ, the EBV-infected autoreactive B cells would produce pathogenic autoantibodies and provide costimulatory signals for the survival of autoreactive T cells [155]. Pender also indicates a role for vitamin D deficiency in this model [155]. As vitamin D (and the vitamin D receptor) has been found to be reduced in patients with autoimmune disease, and reduced vitamin D is noted in higher latitudes which have higher rates of autoimmune disease, a role for vitamin D deficiency in autoimmunity is suspected. Pender suggests that decreased vitamin D aggravates the deficiency in CD8+ T cells, therefore causing further impairment of EBV control [155]. Despite the need for extensive investigation, this hypothesis highlights the intriguing interplay between genetic susceptibility and infection which may play a role in the development of autoimmune disease.

## 11. Conclusions

It appears that EBV has the potential to induce autoimmune responses and indeed autoimmune disease. A wealth of experimental data has been provided over the years in various autoimmune diseases, mainly autoimmune rheumatological conditions. However, the data obtained in autoimmune liver diseases are scarce and more work needs to be done. Also, prospective studies are needed to investigate whether EBV infection is causally related with the development of extrahepatic autoimmune diseases such as Sjögren's syndrome, autoimmune thyroiditis, and rheumatoid arthritis, which frequently cooccur in patients with PBC or AIH. As it stands, the thesis that EBV is strongly linked to autoimmune liver diseases is not supported by convincing data.

## Figures and Tables

**Table 1:** Evidence is support and against a role of Epstein-Barr virus (EBV) in autoimmune liver disease (AiLD). Evidence in support of EBV in the pathogenesis of autoimmune hepatitis (AIH) is largely based around case reports noting the development of AIH following EBV infection. In contrast, studies on EBV and primary biliary cirrhosis (PBC) have been based on the detection of EBV genetic material in PBC patients. As well, molecular mimicry between EBV and self-proteins has also been indicated, with mixed results. Although convincing, it should be noted that EBV is ubiquitous in a large percentage of the population, and thus cannot be causally linked. Evidence linking EBV with PSC is weak, based only on indirect evidence of the role of EBV in the pathogenesis of ulcerative colitis, which is present in many patients with primary sclerosing cholangitis (PSC).

Disease	Evidence in support of EBV	Evidence against EBV
Autoimmune hepatitis (AIH)	(i) Several case reports indicating the development of AIH following EBV infection	(i) The high prevalence of EBV would indicate a higher development of AIH post-EBV infection, which is not the case (ii) Strong EBV links with autoimmune diseases, such as systemic lupus erythematosus (SLE) that are not commonly concomitant with AIH (iii) A CYP2D6_259–271_ mimicking peptide from BVRF1 EBV is totally unreactive in anti-CYP2D6_254–271_ positive patients with AIH-2
	(ii) Serological evidence of EBV infection in AIH patients	
	(iii) Strong link between EBV and rheumatoid arthritis, autoimmune thyroid, and Sjögrens syndrome, which are often concomitant in patients with AIH or other AiLDs	
	(iv) Molecular mimicry and immunological cross-reactivity implicating an EBNA EBV epitope and the immunodominant CYP2D6_259–271_ epitope is demonstrated in patients with AIH-2	

Primary biliary cirrhosis (PBC)	(i) Increased EBV DNA found in peripheral blood mononuclear cells, liver and saliva of PBC patients	
	(ii) Increased EBV early antigen titers in PBC	(i) Increased EBV early antigen titers also found in other autoimmune diseases
	(iii) Strong link between EBV and rheumatoid arthritis, autoimmune thyroid, and Sjogrens syndrome, which are often concomitant in AiLD	(ii) Strong EBV links with autoimmune diseases, such as SLE, that are not commonly concomitant with PBC
	(iv) Homologous regions between EBV proteins and sp100	

Primary sclerosing cholangitis (PSC)	(i) Increased EBV infected B lymphocytes in the intestinal mucosa of ulcerative colitis patients (which is often concomitant in PSC)	(i) No direct evidence or case reports linking EBV to PSC

**Table 2:** Regional amino acid similarity between human cytochrome p450 IID6 (CYP2D6)_257–271_ (the immunodominant epitope of anti-liver kidney microsomal antibodies type 1 in patients with autoimmune hepatitis type 2, and viral sequences originated from hepatitis C, herpes virus 1 (HSV-1), and Ebstein-Barr virus (EBV)). Amino acids (aa) in single letter code. Identical amino acids between human and viral sequences are indicated in bold.

Species	Protein	Code																			
Hepatitis C virus (HCV)	Non structural protein 5B	NS5B							P	*P*	G	D	P	*P*	Q	P	E	Y	D	L	
Hepatitis C virus (HCV)	Envelope 1	E1		*I*	**T**	G	**H**	**R**	**M**	***A***	**W**	D	M	M	M						
Human	Cytochrome P450IID6	CYP2D6	aa254	L	T	E	H	R	M	T	W	D	P	A	Q	P	P	R	D	L	271
Herpes simplex virus type 1 (HSV-1)	Immediate early 175 kDa	IE175						L	S	*P*	R	P	**P**	**A**	**Q**	**P**	**P**	**R**	*R*	R	
Epstein-Barr virus (EBV)	Nuclear protein EBNA	EBNA						P	P	*P*	A	A	**P**	**A**	**Q**	**P**	**P**	**P**	G	*V*	
Epstein-Barr virus (EBV)	Virion protein BVRF1	UL25						*H*	*V*	L	*I*	D	**P**	**A**	R	L	**P**	**R**	*D*	T	

## Abbreviations

AIH: Autoimmune hepatitis
AiLD: Autoimmune liver disease
AMA: Antimitochondrial antibodies
ANA: Antinuclear antibodies
AT: Autoimmune thyroiditis
EBV: Epstein-Barr Virus
GWAS: Genome wide association studies
IBD: Inflammatory bowel disease
IDDM: Insulin dependent diabetes mellitus
MS: Multiple sclerosis
PBC: Primary biliary cirrhosis
PDC: Pyruvate dehydrogenase complex
PSC: Primary sclerosing cholangitis
RA: Rheumatoid arthritis
SjS: Sjogren's syndrome
SLE: Systemic lupus erythematosus
SSc: Systemic sclerosis
UC: Ulcerative colitis.
